# Dissociative experiences and self-reported interoceptive traits in maladaptive daydreamers: a network analysis

**DOI:** 10.1007/s00406-026-02296-w

**Published:** 2026-06-23

**Authors:** Ilaria Bufalari, Lilya Abergel, Andrea Zagaria, Laura Fusar-Poli, Laura Ferreri, Marco Sperduti

**Affiliations:** 1 Department of Developmental and Social Psychology, Sapienza University of Rome, Rome, Italy; 2 Institut Jean Nicod (UMR 8129), Université Paris Sciences et Lettres, Paris, France; 3 Department of Brain and Behavioral Sciences, University of Pavia, Pavia, Italy; 4 Dipartimento di medicina dei sistemi, Università degli studi di Roma Tor Vergata, Via Montpellier, 1, 00133 Rome, Italy; 5 Istituti Clinici Scientifici Maugeri IRCCS, Pavia, Italy; 6 Laboratoire Mémoire, Cerveau et Cognition, Psychology Institute, University Paris Cité, Paris, France

**Keywords:** Maladaptive daydreaming, Dissociation, Absorption, Interoception

## Abstract

Maladaptive Daydreaming (MD) is a proposed mental health condition characterized by compulsive, immersive daydreaming that interferes with daily functioning. Although MD is strongly associated with dissociative experiences, its relation to interoception remains unexplored. In this study, we investigated dissociative symptoms and self-reported interoception in individuals with MD compared to matched controls. A total of 137 Italian-speaking participants (83 MDers and 54 controls) completed an online survey assessing maladaptive daydreaming (MDS-16), dissociation (DES-II), and interoception (MAIA-2). As expected, MDers showed significantly higher levels of dissociation - particularly in the domains of absorption/imagination and depersonalization/derealization - compared to controls. Moreover, MDers exhibited lower scores on specific interoceptive subscales: Attention Regulation, Body Listening, and Trusting. Network analyses highlighted maladaptive daydreaming as a central node linking dissociation and interoception. These findings suggest that individuals with MD may experience altered interoception and that maladaptive daydreaming might function as an escape from unpleasant bodily states. This is the first study to document altered interoception in MD, future research could extend the findings by incorporating objective measures. Clinically, the results highlight the potential utility of interventions aimed at improving interoception - such as mindfulness-based approaches - in addressing maladaptive daydreaming and its dissociative correlates.

**Supplementary Information:**

The online version contains supplementary material available at 10.1007/s00406-026-02296-w.

## Introduction

Maladaptive daydreaming (MD) has been proposed as a clinical syndrome [1] and is characterized by compulsive immersion in complex and vivid imaginary scenarios accompanied by intense emotional reactions and often stereotyped movements [2], leading individuals to create detailed and complex fantasies that come to replace or interfere with their academic activities, interpersonal relationships, and professional life [3].

Individuals suffering from MD may spend up to half of their waking hours immersed in parallel dimensions that are difficult to control [4]. The estimated prevalence of MD is around 2.5% in the general population, although it seems to be higher in young adults [5].

This condition is not yet recognized in diagnostic manuals, but appears to be associated with other psychiatric or mood disorders such as obsessive-compulsive disorder and reinforcement processes similar to those observed in addictions [6]. More recently, MD has been associated with symptoms of dissociation [7, 8]. Indeed, the two constructs are phenomenologically and quantitatively associated. From a phenomenological perspective, both MD and dissociative absorption are characterized by a strong vividness of visual imagery, where the content is experienced with a strong sense of presence. From a quantitative point of view, previous studies reported a high prevalence of MD in patients with dissociative disorders, and vice versa [7, 8]. Moreover, individuals with MD reported higher total scores of the Dissociative Experiences Scale (DES) compared to a control group, as well as in the depersonalization-derealization and dissociative absorption subscales [2, 7, 9, 10]. Thus, while MD could be described as an excessive form of imaginative absorption, i.e. an “absorption disorder” [8], often considered as non-pathological form of dissociation, accumulating evidence suggests that MD might be characterized by more severe symptoms of dissociations, like depersonalization and derealization. In line with this, a recent position paper propose to classify MD as a dissociative disorder in diagnostic manuals [1].

Dissociative disorders are described as “disruption of and/or discontinuity in the normal integration of consciousness, memory, identity, emotion, perception, body representation, motor control, and behavior” [11]. Among dissociative disorders, the DSM-5-TR comprises dissociative identity disorder, dissociative amnesia and depersonalization/derealization. More specifically, depersonalization/derealization is a syndrome characterized by clinically significant persistent or recurrent depersonalization (i.e., experiences of unreality or detachment from one’s mind, self, or body) and/or derealization (i.e., experiences of unreality or detachment from one’s surroundings). While dissociative symptoms are central to dissociative disorder, they may also be clinically relevant in other conditions such as post-traumatic stress disorder, acute stress disorder, borderline personality disorder, panic attacks and functional neurological disorder. Interestingly, recent work has proposed a causal link between altered bodily self-awareness and dissociation, highlighting a central role for interoceptive processes in this relationship [12, 13].

Interoception refers to the perception and integration of the body’s physiological signals [14]. Early accounts organized interoceptive processing into three broad levels [15]: the neural processing of afferent signals from internal organs; the perceptual, affective, and cognitive impact of this neural representation on downstream processes; and the conscious perception of bodily states. Within this third level, Garfinkel et al. [16] proposed an influential distinction between interoceptive accuracy (the objective precision in detecting internal signals), interoceptive sensibility (the self-reported dispositional tendency to focus on bodily states), and interoceptive awareness (the metacognitive correspondence between objective and subjective performance). More recently, Suksasilp and Garfinkel [17] extended this framework into a comprehensive multi-dimensional model spanning distinct processing levels: from bottom-up afferent signal strength to top-down processes of attention and interpretation. A conceptually complementary model proposed by Desmedt et al. [18] organises interoceptive constructs hierarchically according to their level of specificity, identifying four broad factors: interoceptive attention, interoceptive sensing, interoceptive interpretation, and interoceptive memory, each decomposable into more specific subfactors. Interoceptive attention encompasses the capacity to orient, sustain, and regulate attentional resources toward bodily sensations, as well as the tendency not to divert attention away from uncomfortable signals. Interoceptive sensing refers to the conscious and non-conscious registration of bodily signals, including their detection, perceived intensity, and localization. Interoceptive interpretation denotes higher-order, belief-based appraisal of bodily states, including trust in bodily sensations, emotional awareness of their significance, and the degree to which they provoke worry. A critical advantage of this hierarchical framework is that it does not conflate interoceptive dimensions with measurement modalitities: a given factor such as interoceptive attention may be indexed both by objective behavioural measures (e.g., attentional bias tasks) and by self-report questionnaires. This distinction is consequential, because self-report interoception measures do not converge on a single underlying construct of interoceptive sensibility, indeed latent factor analyses reveal that existing questionnaires tap into distinct, loosely related phenomena [19, 20]. In the remainder of this paper, we map the MAIA-2 subscales and associated findings onto the three empirically most relevant factors of this framework - interoceptive sensing, attention, and interpretation - to ensure precise alignment between theoretical constructs and measured outcomes.

Interestingly, several studies have shown associations between dissociative disorders and different components of interoception, although with contrasting findings. While some studies reported altered interoceptive accuracy linked to dissociation [21–23], other showed spared performances [24–26]. Only two studies investigated interoceptive awareness comparing patients suffering from functional seizures [21] or patients with traumatic childhood experiences [27] with healthy controls. Both studies reported higher dissociative experiences in the group of patients. Nevertheless, one study reported lower interoceptive awareness in patients [21], while the second found similar performance between the two groups [27]. The heterogeneity of these findings is, however, unsurprising given the well-documented psychometric limitations of the tasks involved: heartbeat counting task performance is substantially contaminated by time estimation ability and heart rate knowledge [28], the two most widely used cardiac tasks share minimal variance [29], and the construct of interoceptive accuracy as operationalised by these paradigms has been argued to lack the validity needed to support replicable psychological associations [30]. Taken together, these considerations provide both an explanation for the inconsistent evidence and a strong methodological rationale for the self-reported interoceptive approach adopted in the present study.

Self-reported interoceptive processes have been more consistently associated with dissociation than objective measures. Across studies, the most robust pattern is a negative association between dissociation and adaptive interoceptive constructs [18, 20]. Within interoceptive attention, negative associations have been reported for the MAIA subscales Not-Distracting [25, 31], Attention Regulation [32], and Self-Regulation [25]. This pattern collectively suggests that higher dissociation co-occurs with greater habitual avoidance of aversive bodily sensations and reduced capacity to volitionally regulate and sustain attentional engagement with the body. Within interoceptive interpretation, the MAIA subscales Trusting has been negatively associated with dissociation in the majority of studies [25, 33], consistent with the estrangement from bodily experience that characterizes depersonalization and derealization; a discrepant positive finding reported by [32] likely reflects the acute, pharmacologically induced nature of dissociation in that specific sample. A minority of studies reported positive associations with the MAIA subscales Noticing, Emotional Awareness, and Body Listening [31], which may reflect a hypervigilant somatic profile in certain absorptive dissociative presentations rather than genuinely enhanced interoceptive functioning. Evidence from non-MAIA instruments is broadly convergent but requires cautious interpretation. Demartini et al. [34] reported a negative association between dissociation and the Interoceptive Awareness subscale of the Eating Disorder Inventory (EDI). Critically, this subscale measures difficulty in recognising hunger and satiety signals, a maladaptive interoceptive sensing construct, and may reflect, in this contest, a hypervigilant attitude like the positive association reported with some MAIA subscale. Finally, negative associations between dissociation and broader subjective body connectivity indices [35] (Schmitz et al., 2021) provide additional convergent evidence without permitting subfactor-level differentiation. In sum, the existing literature most consistently implicates reduced adaptive interoceptive attention and compromised interoceptive interpretation as the self-reported dimensions most robustly associated with dissociative symptomatology.

To resume, there is substantial empirical evidence of an association between dissociative symptoms and altered interoceptive processes. While the association between dissociation and MD has been supported by different lines of research, to our knowledge, no study investigated interoception in MD. To fill this gap, we assessed dissociative symptoms and self-reported interoception via an online survey in a group of MD and a matched control group.

Based on this evidence, the aim of this work was threefold. First, we wanted to replicate previous findings concerning the link between MD and dissociation. In line with earlier studies, we expected to find higher scores of dissociative symptoms in MD than in control participants. Second, we aimed to test self-reported interoception in MD. Given the established link between MD and dissociative disorders, we expected to find lower self-reported interoceptive attention and interpretation in MD than controls. Third, we aimed to exploratorily investigate, by means of network analysis, the link between interoception, dissociative experiences and MD severity.

## Methods

### Participants

A total of 191 participants, all native Italian speakers, completed the online survey. Participants were enrolled through announcements on the website of the association Maladaptive Daydreaming Italia (https://www.maladaptivedaydreamingitalia.com/), and social networks. All participants voluntarily took part in the study and provided informed consent by selecting the relevant option on the online survey form prior to accessing the questionnaires. The study has been approved by the local Ethical Committee (Department of Brain and Behavioral Sciences, University of Pavia, Project Code 126/23), and was conducted in accordance with the Helsinki Declaration, Convention of the Council of Europe on Human Rights and Biomedicine.

The final sample was composed of 137 participants, 83 MDers (mean age 30.32 ± 9.38 years; 67 females, 10 males, 4 non-binary and 2 who identified as other) and 54 CT (mean age 33.72 ± 11.83 years; 37 females, 15 males, 1 non-binary and 1 who identified as other). The sample was the same as described in Ferreri et al. [36]. For details of screening procedure and exclusion criteria see also Supplementary Material. A post-hoc sensitivity power analysis conducted using G*Power indicated that the present sample size (MD: *n* = 83; Control: *n* = 54) provided 80% power to detect effects of approximately d = 0.49 or larger at α = 0.05 (two-tailed), corresponding to medium-sized effects according to Cohen’s conventions.

The two groups did not differ in age, t(131) = 1.837, *p* = 0.068, and in gender ratio, Chi2 = 5.913, *p* = 0.116. Thirty-three MDers (33.759%) and 8 controls (14.815%) declared having had antecedents of psychiatric or mood disorder, and this difference in percentage was statistically significant. In particular, MDers more frequently declared suffering of several psychiatric conditions (depression, anxiety, obsessive-compulsive disorder, post-traumatic stress disorder) including dissociative disorders (DD). A detailed description, and statistical comparisons of the two groups is presented in Supplementary Table 1.

### Materials

The Maladaptive Daydreaming Scale (MDS-16, [37] Italian version [38] is a 16-item self-report measure assessing the severity of maladaptive daydreaming (11-point Likert scale, 0%–100% in 10% intervals per item). The score is computed as the mean across of all items (range: 0–100), with higher scores indicating greater MD severity. The Italian version demonstrated adequate factorial validity and good convergent and discriminant validity in an Italian community sample [38]. We computed the total score, and the score for the two subscales described in the Italian validation: sensory–motor retreat, capturing the use of rhythmic or sensory–motor strategies (such as pacing, vocalizing, repetitive movements, or music listening) that help maintain absorption in daydreaming; and interference with life, reflecting the maladaptive impact of this immersive fantasy activity on daily functioning. In the present sample, internal consistency was excellent for the total score (Cronbach’s α = 0.951), and interference with life (Cronbach’s α = 0.958) subscale, and good for sensory–motor retreat (Cronbach’s α = 0.875).

The Dissociative Experiences Scale - II (DES-II, [39], Italian version [40] is a 28-item self-report measure of dissociative symptomatology (percentage scale, 0–100 per item). Scores for each item represent the estimated percentage of time the respondent experiences the described phenomenon. We computed the total score as the mean of all items, as well as three subscale scores: Amnesia (experiences of memory gaps and blackouts), Absorption/Imagination (absorption in imaginative activity and alterations in self-perception), and Depersonalization/Derealization (experiences of detachment from oneself or one’s surroundings), with higher scores on each indicating greater severity of the corresponding dissociative experience. The Italian version demonstrated adequate reliability and validity in Italian adolescent and adult samples [40]. In the present sample, internal consistency was excellent for the total score (α = 0.934) and good for all three subscales (Amnesia: α = 0.856; Absorption/Imagination: α = 0.863; Depersonalization/Derealization: α = 0.856).

The Multidimensional Assessment of Interoceptive Awareness,* Version 2* (MAIA-2, [41, 42], Italian version [43] is a 37-item self-report instrument assessing multiple facets of self-reported interoceptive processing (6-point Likert scale, 0–5 per item). Each subscale score is computed as the mean of its constituent items, yielding scores ranging from 0 to 5. Before computing subscale means, items belonging to the Not-Distracting subscale (items 5, 6, 7, 8, 9, 10) and the Not-Worrying subscale (items 11, 12, 15) are reversed such that higher scores on all subscales consistently reflect greater endorsement of the respective interoceptive tendency in its adaptive direction. The following subscale were computed: Noticing (awareness of uncomfortable, comfortable, and neutral body sensations; α = 0.727); Not-Distracting (tendency not to ignore or distract oneself from sensations of pain or discomfort; α = 0.803); Attention Regulation (ability to sustain and control attention to body sensations; α = 0.882); Self-Regulation (ability to regulate distress by directing attention to body sensations; α = 0.774); Not-Worrying (tendency not to experience emotional distress with sensations of pain or discomfort; α = 0.768); Emotional Awareness (awareness of the connection between body sensations and emotional states; α = 0.868); Body Listening (active listening to the body for insight; α = 0.838); and Trusting (experience of one’s body as safe and trustworthy; α = 0.853). The Italian version demonstrated adequate internal consistency and structural validity [43]. In the present sample, internal consistency was excellent for the total score (α = 0.906) and ranged from acceptable to good across subscales (range: 0.727–0.882; all α > 0.70).

### Procedure

The study was conducted completely on-line via the open-access software Psytoolkit [44, 45]. Participants were firstly informed of the academic nature of the study, and they read and accepted an informed consent. Then, they responded to some socio-demographic questions, and completed the above mentioned questionnaire, along with other questionnaires that are reported elsewhere [36]. Indeed, the data presented here are part of a larger survey on the role of music in modulating daydreaming experiences. The complete survey is freely available here: https://osf.io/5wj8t/files/osfstorage.

### Data analysis

Data processing and analyses were performed in R [46] using the tidyverse ecosystem [47] and the plyr [48], bootnet [49], qgraph [50], glasso [51], and networktools [52] packages.

Independent-sample t-tests were implemented to compare MDers and control participants on demographic and relevant variables (DES-II, MAIA-2). For t-tests, Cohen’s d was used to report effect size.

Furthermore, to exploratively visualize the conditional dependencies between MDS, MAIA-2 and DES-II scores, a Gaussian Graphical Model (GGM) was estimated on the whole sample by applying the graphical least absolute shrinkage and selection operator regularization.

(gLASSO, [53] in combination with the Extended Bayesian Information Criterion model selection (EBIC; [54]; hyperparameter gamma = 0.50). To quantify the extent to which MDS was conditionally connected to both the MAIA and DES domains, bridge strength centrality was computed [55]. The stability of this metric was assessed using a case-dropping bootstrap procedure based on 1,000 resamples, from which the correlation stability coefficient was derived [49]. Within the GGM, nodes represent observed variables connected by undirected edges. These edges reflect partial correlation coefficients, capturing the strength of conditional associations between pairs of variables while controlling for the influence of all other variables in the network. The network structure was visualized using the Fruchterman-Reingold algorithm [56], where positive associations are depicted in green, negative associations in red, and the thickness of each edge corresponds to the magnitude of the partial correlations.

## Results

### Between groups comparison

Prior to conducting between-group comparisons, statistical assumptions were formally assessed for all 15 outcome variables. Normality was evaluated using the Shapiro–Wilk test, applied separately to each group, Homogeneity of variance was assessed using Levene’s test (Brown-Forsythe variant). Results indicated that normality was not violated for the majority of MAIA-2 subscales in either group. However, significant departures from normality were observed for almost all DES-II subscales, and the MDS-16 total score and subscales. Significantly unequal variances were found for five variables: MAIA Not-Distracting, Self-Regulation, Absorption/Imagination, Depersonalization/Derealization, and MDS-16 total score. Full results are reported in Supplementary Table 2.

Therefore, between-group comparisons were conducted using Welch’s t-tests, which does not assume homogeneity of variance and has been shown to perform comparably than Student’s t-test across a range of conditions, including those involving unequal group sizes and heterogeneous variances [57]. Given the number of independent comparisons performed (*n* = 15), a Bonferroni correction was applied to control for family-wise error rate [58], yielding an adjusted significance threshold of α = 0.003 (0.05/15). In the following, we only report statistically significant differences. Descriptive statistics (mean and standard deviation) for all variables by group, and complete Welch’s t-test statistics are reported in the Supplementary Table 3.

*Maladaptive Daydreaming Scale*: MDers (M = 73.14, SD = 15.20) had higher scores on the MDS total score compared to controls (M = 31.59, SD = 22.79), *t*(83.59) = 11.802, *p* < 0.001, *Cohen’s d* = 2.240. Somato-sensory retreat (MD: M = 71.94, SD = 19.13; Control: M = 42.59, SD = 22.31; *t*(100.9) = 7.950, *p* < 0.001, *Cohen’s d* = 1.436), and Interference with life (MD: M = 74.34, SD = 15.88; Control: M = 20.58, SD = 26.74; *t*(77.5) = 13.325, *p* < 0.001, *Cohen’s d* = 2.581) showed the same pattern.

*The Dissociative Experiences Scale*: MDers had higher scores (M = 29.04, SD = 18.12), compared to controls (M = 19.97, SD = 13.70), on the DES-II total score, *t*(131.88) = 3.327, *p* = 0.001, *Cohen’s d* = 0.549, and the Absorption/Imagination subscale (MD: M = 45.06, SD = 21.38; Control: M = 30.37, SD = 17.69), *t*(127.3) = 4.369, *p* < 0.001, *Cohen’s d* = 0.734. The differences in the Depersonalization/Derealization was significant (*p* = 0.007), but not survived correction, while the difference in the Amnesia subscale was not significant.

*Multidimensional Assessment of Interoceptive Awareness, Version 2*: We found significant differences, with lower scores for MDers compared to controls, in the following subscales: Body Listening, MD (M = 29.04, SD = 18.12) vs. Control (M = 19.97, SD = 13.70), *t*(102.88) = −3.123, *p* = 0.002, *Cohen’s d* = 0.561; and Trusting, MD (M = 1.97, SD = 1.41) vs. Control (M = 2.66, SD = 1.26), *t*(121.85) = −2.986, *p* = 0.003, *Cohen’s d* = 0.510. The same trend was observed for Attention Regulation (*p* = 0.007), but the difference did not survive correction for multiple comparisons. No significant differences were reported for the remaining subscales.

### Partial correlation network

**Fig. 1:**
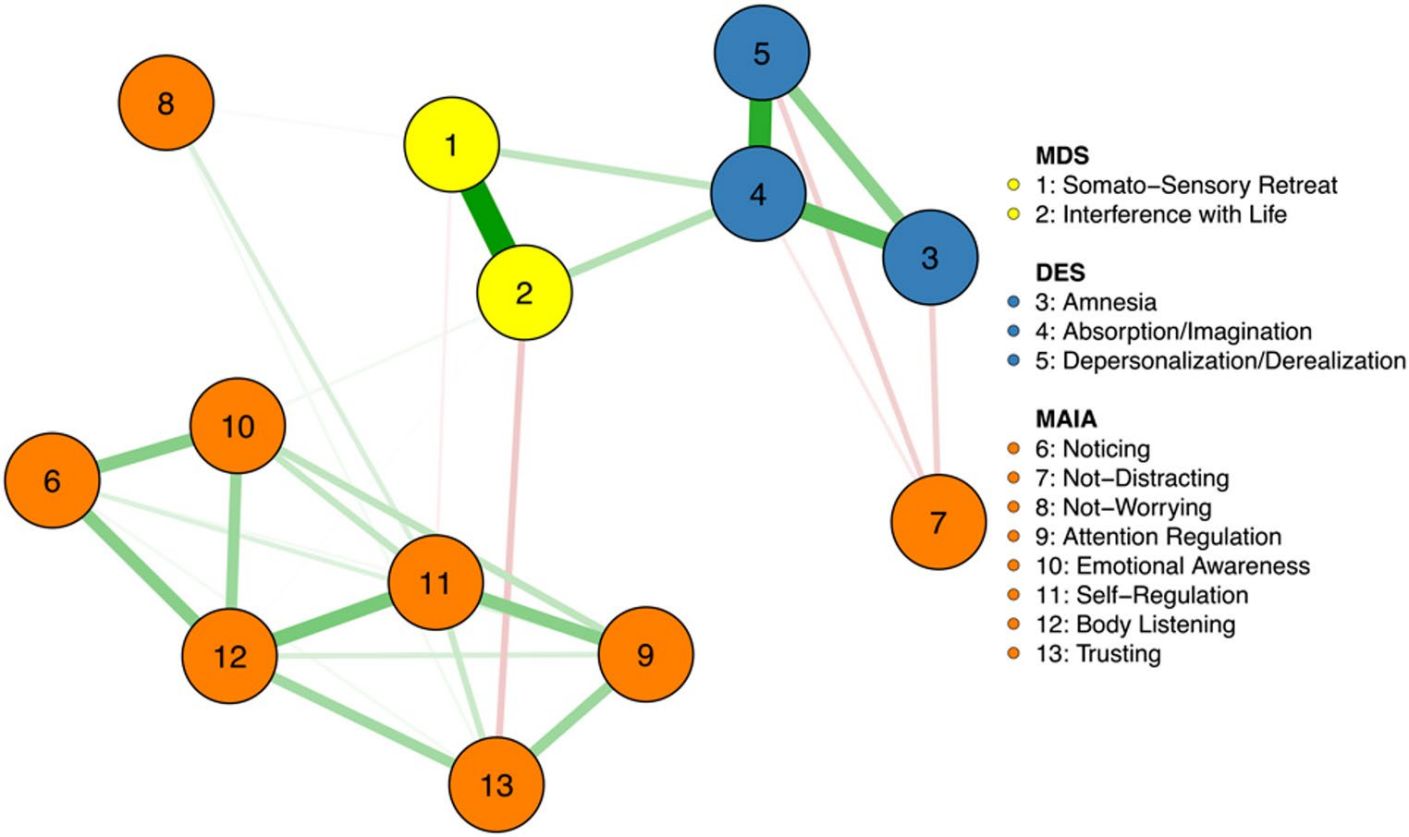
EBICglasso network

Figure 1 displays the network estimated using the EBICglasso procedure. The resulting structure was relatively sparse, with 30 out of 78 possible edges (38.5%) estimated to be higher than zero. The average edge weight was 0.144, with the corresponding edge weight matrix reported in Supplementary Table 4.

**Fig. 2:**
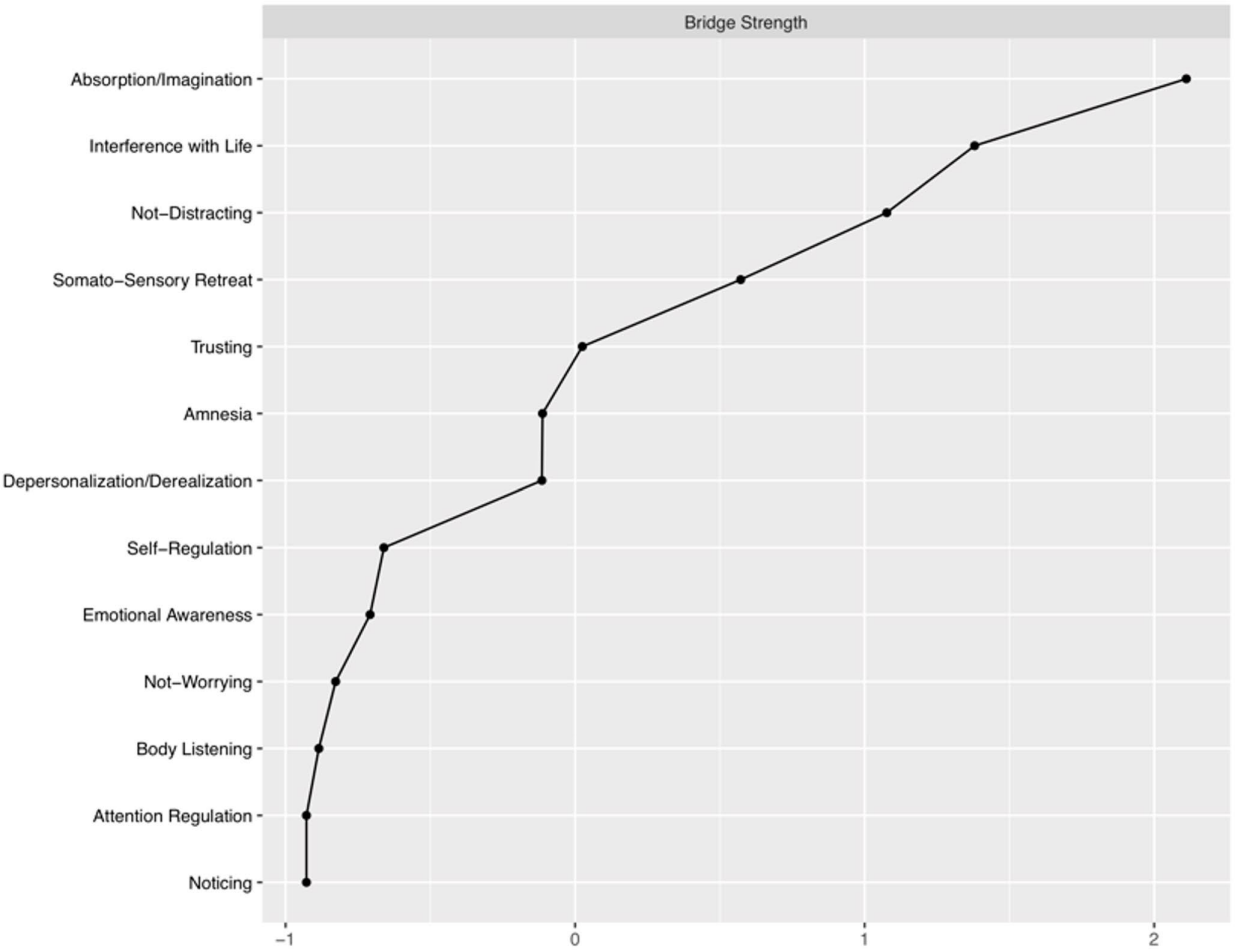
Bridge strength centrality estimates

As highlighted by the bridge strength analysis (Fig. 2), which showed satisfactory stability (CS coefficient = 0.438; [49]), the MDS subscale Interference with Life emerged as one of the key bridging nodes, linking core dimensions of the DES with the MAIA subscales. Notably, the MAIA subscale Not-Distracting also displayed notable cross-domain connectivity, particularly with dissociative components, but remained disconnected from other interoceptive nodes - suggesting a distinct mechanism.

More specifically, the MDS subscale Interference with Life showed a positive association with Absorption/Imagination (partial *r* = 0.147) and a negative association with Trusting (partial *r* = − 0.107). Further associations were observed between the MDS subscale Somato-Sensory Retreat and Absorption/Imagination (partial *r* = 0.127), as well as Self-Regulation (partial *r* = − 0.030) and Not−Worrying (partial *r* = − 0.011). As expected, the two MDS nodes were strongly interconnected (partial *r* = 0.503).

Interestingly, the MAIA subscale Not-Distracting did not exhibit any associations with other MAIA subscales. Instead, it showed negative associations with Amnesia (partial *r* = − 0.091), Absorption/Imagination (partial *r* = − 0.042), and Depersonalization/Derealization (partial *r* = − 0.091). In contrast, the other MAIA dimensions were closely interconnected within the network, as were the core components of the DES, reflecting several intra-domain associations (see Supplementary Table 4).

## Discussion

The aim of our study was to replicate previous findings on the link between MD and dissociation, and to investigate, for the first time, interoceptive traits in participants experiencing MD. We hypothesized that dissociative experience, as measured by the DES-II, may be more pronounced in MD. Additionally, given the general negative relationship between dissociation and interoception, we expected that MD would report lower interoceptive traits as assessed by the MAIA-2. We also exploratorily tested the interplay between interoception, dissociation and the severity of maladaptive daydreaming with network analysis.

In line with our hypotheses, we found that MD participants, as compared to controls, reported higher total scores at the DES-II, as well as higher absorption/imagination and derealization/depersonalization subscores, even if difference in the latter did not survive correction for multiple comparisons. Conversely, the two groups were comparable on the amnesia score. These findings are coherent with previous reports and confirm the association between MD and dissociation [1, 37, 38, 59]. The differential profile across DES-II subscales is interesting and theoretically informative. The strongest group difference was observed for absorption/imagination, which is consistent with the conceptualisation of MD as an absorption disorder [8]: compulsive immersion in vivid imaginary scenarios is both a defining feature of MD and the phenomenological substrate of dissociative absorption, rendering this dimension the most direct point of overlap between the two constructs. Elevated depersonalization/derealization in MD may reflect the cumulative effect of chronically withdrawing attention from bodily and environmental reality, a process that, over time, may erode the stability of reality monitoring and self-continuity, generating experiences of unreality and self-estrangement [1]. By contrast, the absence of group differences in amnesia is consistent with the view that MD is specifically linked to the absorption-derealization spectrum of dissociation rather than to the structural-amnesic spectrum characteristic of more severe trauma-related dissociative disorders, further supporting the proposal that MD represents a distinct dissociative subtype [1].

Crucially, in line with our main hypothesis, MD was associated with reduced self-reported interoception, as evidenced by significantly lower scores on Body Listening and Trusting, and a trend toward lower Attention Regulation scores that did not survive Bonferroni correction. Interpreted within the hierarchical framework of Desmedt et al., (2023), Body Listening and Trusting are subfactors of interoceptive interpretation: they capture, respectively, the active disposition to consult bodily sensations as a source of affective insight, and the experience of one’s body as safe and trustworthy. Attention Regulation, by contrast, is a subfactor of interoceptive attention: it reflects the capacity to sustain and volitionally direct attentional resources toward bodily sensations. The present findings are best understood as indicating selective deficits in higher-order interoceptive interpretation and attentional regulation in MD, consistent with the broader literature reporting lower interoceptive attention and interpretation scores in populations with elevated dissociative symptomatology, including functional neurological disorder [25] and trauma-exposed samples [27], and with studies specifically reporting lower Attention Regulation [32] and Trusting [25, 33] in association with dissociation.

An important contextual factor in interpreting the between-group differences in interoceptive interpretation is the higher rate of psychiatric comorbidities reported by the MD group relative to controls. As reviewed by Khalsa and Verdonk [60], depression, anxiety, OCD, PTSD and dissociative disorders - all of which were more frequent in MD than controls in our sample - are each associated with interoceptive atypicalities. The reduced Body Listening and Trusting observed in the MD group may therefore reflect, at least in part, the cumulative interoceptive consequences of these co-occurring conditions rather than, or in addition to, MD itself. Nevertheless, these findings remain theoretically meaningful within the present framework: regardless of their proximal cause, deficits in interoceptive interpretation constitute a plausible mechanism sustaining maladaptive daydreaming across multiple psychiatric contexts, insofar as they increase the aversiveness of bodily experience and thus the motivational pull toward fantasy as a regulatory escape. Future research should examine whether these interoceptive profiles vary as a function of specific comorbidity patterns, and whether they mediate the relationship between psychiatric symptom severity and MD engagement across diagnostic groups.

Given the cross-sectional design of our study, it is not possible to draw any causal inference. On the one hand, MD may be the cause of interoceptive alterations. Indeed, being compulsively immersed in vivid fantasies could reduce the ability to focus on bodily sensation, and, in turn, bodily sensation would be less precise (untrustworthy) and less available to gather insights on one’s effective state. On the other hand, MD may be a consequence of feeling one’s body as untrustworthy. It has been shown that daydreaming could function as a distraction from an unpleasant reality [61]. Thus, distracting from an untrustworthy body via inner fantasies would lead to disengage attention from bodily sensation, and not use these sensations for insights. We acknowledge that these hypotheses are highly speculative, and that more studies are warranted to directly test them. Predictive processing accounts of interoception provide a theoretical framework within which the observed associations can be more precisely contextualised. Within these frameworks, the brain continuously generates and updates top-down predictions about the physiological state of the body, a process serving allostasis, against which incoming bottom-up interoceptive signals are compared (Quigley et al., 2021; Jungilligens et al., 2022). The sense of body ownership and self-continuity emerges when top-down models and bottom-up signals are well aligned; when they are chronically mismatched, or when incoming interoceptive signals are systematically down-weighted by imprecise predictions, experiences of unreality, detachment, and loss of body ownership may arise (Jungilligens et al., 2022). Elevated interoceptive prediction errors have been empirically associated with greater dissociative symptomatology across diagnostic contexts, including FND (Jungilligens et al., 2022) and migraine (Koreki et al., 2025), supporting the transdiagnostic relevance of this mechanism. Crucially, our results show restricted difference to Body Listening and Trusting, both subfactors of interoceptive interpretation (Desmedt et al., 2023). Within predictive processing accounts, interoceptive interpretation could reflect the precision weighting assigned to bodily signals: low interoceptive interpretation indicates that interoceptive signals are chronically down-weighted or experienced as aversive (Jungilligens et al., 2022). The finding that MD is specifically associated with reduced interoceptive interpretation, rather than with reduced attentional capacity per se, is most consistent with a hypothesis in which individuals with MD experience the body as an unreliable or threatening source of information, motivating a retreat into fantasy as an escape from unresolved and aversive interoceptive states [61]. In this account, chronic imprecision in the interoceptive predictive model generates persistent prediction errors that are experienced as somatic discomfort or unreliability; fantasy provides an alternative attentional space free from such errors. Conversely, an alternative hypothesis could be that compulsive immersion in fantasy erodes interoceptive interpretation by depriving the predictive model of the regular bottom-up updating necessary to maintain reliable interoceptive beliefs, though the absence of a significant Attention Regulation difference between MD and control after correction makes this attention-depletion account less directly supported by the present data. The present study cannot adjudicate between these two directions of effect, nor can it test causal sequential pathways (e.g., interoceptive interpretation → maladaptive daydreaming severity → interoceptive attention). Indeed, the cross-sectional design provides no information about the directionality of the proposed relationship between variables: mediation analyses on simultaneously collected self-report observational data can establish statistical associations but not causal relationships or temporal ordering [62]. Future research should employ designs capable of establishing temporal precedence and testing causal mechanisms. Longitudinal studies using cross-lagged panel models could examine, for example, whether low interoceptive trust at baseline prospectively predicts greater MD severity over time, and vice versa (e.g., [62]). Within-person experience sampling designs, capturing daily fluctuations in interoceptive trust and fantasy engagement, would allow within-person mediation analysis that separates state from trait-level mechanisms. Finally, randomised controlled trials of interventions specifically targeting interoceptive interpretation, with MD severity as a primary outcome, would provide the most direct causal test of the hypothesis that reduced interoceptive trust drives and maintains maladaptive daydreaming.

Interestingly, network analysis offered further insight into the relationship between maladaptive daydreaming, dissociation, and interoceptive sensibility. Maladaptive daydreaming, in particular the Interference with life subscale, emerged as the second most prominent bridge node in the network. In particular both MDS subscales served as critical connectors between dissociative and interoceptive domains. This results suggest the association between MD and both dissociation (e.g., absorption/imagination) and interoceptive alterations (e.g., low trust in bodily sensations).

Specifically, the network revealed a dissociation between the functional impairment and sensory-motor dimensions of MD. MDS Interference with Life showed a specific negative connection with Trusting (partial *r* = − 0.107). Within the Desmedt et al. (2025) hierarchical framework, Trusting is a subfactor of interoceptive interpretation - the belief-based experience of one’s body as safe and reliable. The finding that reduced interoceptive trust is specifically linked to the functionally impairing dimension of MD is consistent with the predictive processing account proposed above: when bodily signals are experienced as untrustworthy, the body ceases to function as a reliable regulatory and informational resource, increasing reliance on fantasy with direct functional consequences for daily life. MDS Somato-sensory retreat - reflecting the kinesthetic and sensory-motor features of MD such as pacing, music use, and compulsive urge to return to fantasy - showed (weaker) negative connections with MAIA Self-Regulation (partial *r* = − 0.030) and Not-Worrying (partial *r* = − 0.011). Self-Regulation is a subfactor of interoceptive attention (Desmedt et al., 2025) reflecting the capacity to use body-focused attention as a strategy for regulating psychological distress. This negative association suggests that higher MD severity is linked to a reduced capacity to down-regulate distress through attentive engagement with bodily sensations, consistent with the view that individuals with MD may have diminished access to the body as a regulatory resource, increasing reliance on fantasy as an alternative self-regulatory strategy. Concerning Not-Worrying – a subcomponent of interoceptive interpretation – the results suggests that MD is linked with reduced tendency to not experience emotional distress about sensations. This tentatively suggests that the sensory-motor and immersive dimension of MD is associated with both reduced use of body-focused attention for distress regulation and a reduced tendency to remain non-catastrophizing about bodily sensations. However, given the weakness of these edges, these associations should be treated with considerable caution.

Notably, the MAIA subscale Not-Distracting demonstrated unique connectivity: while it was not intercorrelated with other interoceptive facets, it showed significant negative associations with multiple dissociative symptoms. Not-distracting is the tendency to not ignore unpleasant physical sensation, and seems to be an independent interoceptive factor [19, 63]. This could explain why this facet does not cluster with the others MAIA components. On the other hand, the negative correlation between not-distracting and depersonalization have been previously reported by Bowling et al., [31]. This finding supports the aforementioned hypothesis that dissociation could be driven by escaping from an unpleasant reality, in this case represented by the feelings of pain and discomfort in the body.

The relationship between escaping from physical discomfort, daydreaming and dissociation could represent a downward spiral: the more discomfort is avoided via fantasies, the more dissociative symptoms are important, and the more the individual is disconnected from one’s body. This pattern suggests that the tendency to avoid unpleasant bodily sensations may constitute a distinct interoceptive risk factor for dissociation and MD. These findings underscore the potential of network analysis in identifying key psychological mechanisms and targets for clinical intervention.

We also found that Emotional Awareness- i.e., the awareness of the connection between bodily sensations and emotions - was positively associated with dissociative scores, in particular with absorption/imagination (partial *r* = 0.024). This aligns with previous findings showing a positive link between depersonalization and this facet of interoception [31], and could suggest a heightened affective experience in MD. Such heightened affective reaction in MD could stem from dysfunctional emotional regulation abilities [64, 65].

To our knowledge, this is the first study to specifically analyze the association between interoception and MD. Nevertheless, several limitations must be considered when interpreting the present findings. First, the sample was self-selected and recruited via a specialised MD organisation and associated social networks, which may have resulted in a self-selected sample with heightened awareness of, or identification with, MD-related experiences. This has direct implications for the generalisability of the findings: individuals who actively seek out MD-specific communities may be those experiencing the most pronounced dissociative and interoceptive difficulties. A directly comparable phenomenon has been documented in the aphantasia literature, where self-reported aphantasics displayed more extreme phenotypic profiles than individuals identified via single-blind procedure suggesting that self-identification systematically exaggerates the apparent severity of the condition [66]. An analogous self-identification bias may operate in the present sample, such that the observed between-group differences in dissociation and interoceptive interpretation could be inflated relative to what would be observed in a broader or clinically ascertained MD population. Future research should complement self-report identification by incorporating single-blind procedures to reduce self-referral bias and improve the representativeness of MD samples. Second, interoceptive processing was assessed exclusively via the MAIA-2, which does not capture other interoceptive constructs or sub-dimensions. Findings are therefore restricted to the self-reported aspects of interoception indexed by this instrument. Third, specific limitations apply to the network analysis. The network was estimated on the pooled sample (*N* = 137), which, for a 14-node network, falls well below the sample sizes recommended for stable edge weight recovery. Simulation studies demonstrate that sample size requirements increase rapidly with node count, and that networks of 10–15 nodes typically require several hundred participants under standard conditions to achieve reliable edge recovery and avoid false negatives [67]. The present network structure should therefore be treated as preliminary and hypothesis-generating rather than definitive: individual edge weights may be imprecisely estimated, and absent edges may reflect insufficient power rather than true conditional independence. Additionally, Gaussian Graphical Models identify patterns of conditional dependence among variables but cannot establish causal directionality or mechanistic pathways; network findings complement but do not replace experimental or longitudinal designs. Fourth, the potential confounding role of psychiatric comorbidities. The MD group reported significantly higher rates of depression, anxiety, OCD, PTSD, and dissociative disorders than controls, and these conditions were assessed via self-report without clinical verification. Since each of these disorders is independently associated with atypical interoceptive processing [60], it is not possible to determine from the present data whether the observed differences in Body Listening and Trusting reflect interoceptive features specific to MD, the interoceptive consequences of co-occurring psychopathology, or a combination of both. Future studies should include verified psychiatric assessments to disentangle the independent contributions of MD and mental disorders to the interoceptive profile observed here. A further limitation, linked to the previous point, is that the study did not assess trauma exposure, which is associated with MD [68], dissociative symptomatology [69], and atypical interoceptive processing [70]. Failure to measure or control for trauma means that observed associations among the three domains may partially reflect shared variance with trauma history rather than direct relationships among MD, dissociation, and interoception. Future studies should explicitly assess trauma exposure to disentangle its potential contribution to the observed associations and to determine whether it moderates the relationship between MD severity and interoceptive interpretation deficits.

In conclusion, we demonstrated reduced interoceptive abilities in individuals with MD, and found that some facets of interoception were associated with both dissociative experiences and the severity of MD. The link between daydreaming and interoception deserves further investigation using other methods not restricted to self-reports assessing interoceptive component not covered in the present work.

Our findings could be relevant for psychotherapeutic intervention in MD. Mindfulness-based interventions may be relevant insofar as they cultivate a peculiar relationship with bodily sensations: rather than suppressing, avoiding, or becoming absorbed away from them, individuals are trained to attend to internal signals with openness, curiosity, and non-judgment. Recent meta-analytic evidence indicates that mindfulness-based interventions produce small-to-moderate improvements in self-reported interoception, and that improvements in self-reported interoception are associated with reductions in psychological distress [71]. Approaches such as Mindful Awareness in Body-Oriented Therapy propose that interoceptive skills can be cultivated through progressive training in identifying, accessing, and appraising internal bodily signals, thereby increasing tolerance of uncomfortable sensations and supporting emotion regulation [72]. Interestingly, mindfulness based interventions (MBI) have been shown to parallelly reduce dissociative symptoms and enhance interoceptive abilities [73]. Thus, targeting these maladaptive interoceptive schemas may help individuals with MD to remain engaged with bodily and emotional cues, rather than using daydreaming or dissociative absorption as a self-regulatory strategy. This is coherent with preliminary findings showing positive effects of MBI in reducing MD [74]. Future interventional studies should directly test whether changes linked to MBI in interoceptive attention and interpretation mediate reductions in dissociative symptoms and maladaptive daydreaming. Ultimately, if mindfulness-based approaches targeting interoception and dissociative symptoms prove effective in reducing maladaptive daydreaming, the functional consequences may extend well beyond symptom reduction. Given that MD is associated with significant interference with daily functioning, occupational performance, and social engagement [2, 37], interventions capable of reducing fantasy escape could translate into meaningful improvements in quality of life across both community and clinical populations. This positions interoception-focused interventions not only as a theoretically grounded treatment target for MD, but as a potential pathway toward broader functional recovery in a condition that currently lacks established, evidence-based therapeutic options.

## Supplementary Information

Below is the link to the electronic supplementary material.

Supplementary Material 1

## Data Availability

The data are available from the authors upon reasonable request.
