# Supplementary material for: Dissociative experiences and self-reported interoceptive traits in maladaptive daydreamers: a network analysis
Source: Eur Arch Psychiatry Clin Neurosci. 2026 Jun 23;276(5):2063–75. doi: 10.1007/s00406-026-02296-w (PMC13481386; doi:10.1007/s00406-026-02296-w)
Supplement: Supplementary file 1 [file 406_2026_2296_MOESM1_ESM.docx]

Index

[Screening and exclusion criteria	2](#_Toc229391876)

[Supplementary Table 1. Self-reported psychiatric and mood disorders	4](#_Toc229391877)

[Supplementary Table 2. Assumption checks for between-group Welch’s t-tests.	5](#_Toc229391878)

[Supplementary Table 3. Descriptive statistics and results of Welch's independent-samples t-tests.	6](#_Toc229391879)

[Supplementary Table 4. Edge-weight matrix extracted from the EBICglasso network	8](#_Toc229391880)

# **Screening and exclusion criteria**

Participants were classified into two groups - maladaptive daydreamers (MDers) and controls (CT) - using a two-stage procedure. In the first stage, 191 participants completed a single screening question assessing self-identification as a maladaptive daydreamer (adapted from Somer et al., 2017). In the second stage, all participants completed a custom made self-report version of the Structured Clinical Interview for Maladaptive Daydreaming (SCID-MD; Somer et al., 2017), regardless of their screening response. Specifically, participants were asked to respond "true" or "false" to the following statements: Criterion A: 1) In a consecutive period of 6 months or more I had persistent and recurring vivid and imaginative fantasy activity (daydreams); 2) While daydreaming, I am very absorbed/immersed in fantasies which may have visual, auditory or affective characteristics. Criterion B: 1) Daydreams are triggered, maintained or reinforced by listening to music; 2) Daydreams are triggered, maintained or reinforced by stereotyped movements (e.g. walking, rocking, moving hands); 3) I often daydream when I feel anxious or bored; 4) The duration or intensity of daydreams increases in the absence of other people (e.g., I daydream more when I am alone); 5) I am annoyed when I cannot daydream or when daydreams are interrupted or curbed; 6) I prefer to daydream rather than engage in daily chores, social, academic, or professional activities; 7) I have made repeated unsuccessful efforts to control, reduce, or stop daydreaming. Criterion C: Daydreaming causes me significant discomfort or impairs my social, work, or other important areas of my life. Criterion D: Daydreaming is due to the direct physiological effects of a substance (e.g. a drug, medication) or a general medical condition (e.g. dementia).

Classification as an MDer required satisfying all of the following conditions simultaneously: (1) affirmative response to the screening question; (2) endorsement of both items from Criterion A; (3) endorsement of at least one item from Criterion B; (4) endorsement of Criterion C (daydreaming causes significant distress or functional impairment); and (5) non-endorsement of Criterion D. Participants who did not meet all five conditions were not classified as MDers.

As 35 participants responded affirmatively to the screening question but did not endorse Criterion C, we do not classify these participants as MDer, since this criterion exactly test the maladaptive nature of the fantasizing activity. Moreover, 3 participants met all SCID-MD criteria (A, B, C, not D) but did not endorse the screening question. These 38 participants did not satisfy the full MDer classification requirements and were excluded from all further analyses, as their status precluded unambiguous assignment to either group.

Of the 153 remaining participants, 93 participants were classified as MDers (following the abovementioned five criteria) and 60 participants were classified as controls (CT), because they responded negatively to the screening question and did not meet all SCID-MD A,B,C, D criteria. We note that this group may include individuals who partially endorsed one or more SCID-MD criteria without meeting the full classification.

Moreover, following our preregistered exclusion criteria, 15 participants were excluded since they completed the survey with a duration exceeding ± 2 SD, and 1 for having missed more than 2 attentional check questions. Thus, the final sample was composed of 137 participants, 83 MDers (mean age 30.32 ± 9.38 years; 67 females, 10 males, 4 non-binary and 2 who identified as other) and 54 CT (mean age 33.72 ± 11.83 years; 37 females, 15 males, 1 non-binary and 1 who identified as other).

# **Supplementary Table 1**. Self-reported psychiatric and mood disorders

|  | **MDD group** | **control group** | **Chi^2^** | |
| --- | --- | --- | --- | --- |
|  |  |  | **Value** | **p** |
| **Depression** | 21 (25.301 %) | 4 (7.407 %) | 7.022 | **<0.01** |
| **Bipolar Disorder** | 1 (1.205 %) | 1 (1.852 %) | 0.095 | 0.758 |
| **Anxiety** | 19 (22.892 %) | 4 (7.407 %) | 5.615 | **0.018** |
| **Schizophrenia** | 0 (0 %) | 0 (0 %) | NA | NA |
| **OCD** | 11 (13.253 %) | 1 (1.852 %) | 5.321 | **0.021** |
| **Addiction** | 4 (4.819 %) | 0 (0 %) | 2.681 | 0.102 |
| **Eating Disorder** | 10 (12.048 %) | 2 (3.704 %) | 2.850 | 0.091 |
| **Borderline** | 1 (1.205 %) | 0 (0 %) | 0.655 | 0.418 |
| **ASD** | 2 (2.410 %) | 2 (3.704 %) | 0.193 | 0.660 |
| **ADHD** | 1 (1.205 %) | 2 (3.704 %) | 0.954 | 0.329 |
| **PTSD** | 8 (9.639 %) | 0 (0 %) | 5.528 | **0.019** |
| **DD** | 9 (10.843 %) | 1 (1.852 %) | 3.909 | **0.048** |

OCD = obsessive compulsive disorder; ASD = autistic spectrum disorder; ADHD = attention deficit hyperactivity disorder; PTSD = post-traumatic stress disorder; DD= dissociative disorders

# **Supplementary Table 2**. Assumption checks for between-group Welch’s t-tests.

|  | **Shapiro–Wilk MD group** | | | **Shapiro–Wilk Control group** | | | **Levene's Test (df₁ = 1, df₂ = 135)** | | |
| --- | --- | --- | --- | --- | --- | --- | --- | --- | --- |
| **Variable** | ***W*** | ***p*** | **Normality** | ***W*** | ***p*** | **Normality** | ***F*** | ***p*** | **Equal Variances** |
| ***MAIA-2*** | | | | | | | | | |
| Noticing | 0.979 | *.203* | **OK** | 0.963 | *.095* | **OK** | *0.178* | *.674* | **Yes** |
| Not-Distracting | 0.978 | *.167* | **OK** | 0.991 | *.954* | **OK** | *4.459* | *.037* | **No** |
| Not-Worrying | 0.976 | *.129* | **OK** | 0.964 | *.103* | **OK** | *0.063* | *.802* | **Yes** |
| Attention Regulation | 0.972 | *.067* | **OK** | 0.982 | *.595* | **OK** | *0.014* | *.907* | **Yes** |
| Emotional Awareness | 0.970 | *.051* | **OK** | 0.956 | *.044* | **Violated** | *0.070* | *.792* | **Yes** |
| Self-Regulation | 0.969 | *.039* | **Violated** | 0.952 | *.029* | **Violated** | *6.263* | *.014* | **No** |
| Body Listening | 0.933 | *< .001* | **Violated** | 0.956 | *.045* | **Violated** | *2.156* | *.144* | **Yes** |
| Trusting | 0.932 | *< .001* | **Violated** | 0.974 | *.274* | **OK** | *0.882* | *.349* | **Yes** |
| ***DES-II*** | | | | | | | | | |
| Total score | 0.935 | *< .001* | **Violated** | 0.888 | *< .001* | **Violated** | *3.862* | *.051* | **Yes** |
| Amnesia | 0.820 | *< .001* | **Violated** | 0.845 | *< .001* | **Violated** | *1.568* | *.213* | **Yes** |
| Absorption/Imagination | 0.975 | *.100* | **OK** | 0.940 | *.010* | **Violated** | *5.098* | *.026* | **No** |
| Depersonalization/Derealization | 0.837 | *< .001* | **Violated** | 0.781 | *< .001* | **Violated** | *4.488* | *.036* | **No** |
| ***MDS-16*** | | | | | | | | | |
| Total score | 0.966 | *.028* | **Violated** | 0.859 | *< .001* | **Violated** | *3.989* | *.048* | **No** |
| Somato-sensory retreat | 0.945 | *.001* | **Violated** | 0.940 | *.010* | **Violated** | *0.719* | *.398* | **Yes** |
| Interference with life | 0.968 | *.039* | **Violated** | 0.746 | *< .001* | **Violated** | *3.872* | *.051* | **Yes** |
|  | | | | | | | | | |

Shapiro–Wilk (W) tests were applied separately to each group to assess the normality of the distribution of each outcome variable. ‘OK’ indicates that the null hypothesis of normality was not rejected (p ≥ .05); ‘Violated’ indicates a significant departure from normality (p < .05). Levene’s test (Brown–Forsythe variant, centred on the group median) was used to assess homogeneity of variance; ‘Yes’ indicates that the assumption of equal variances was not rejected (p ≥ .05), and ‘No’ indicates that variances differed significantly between groups (p < .05).

# **Supplementary Table 3**. Descriptive statistics and results of Welch's independent-samples t-tests.

|  | **MD (n = 83)** | | **Control (n = 54)** | |  |  |  |  |  |
| --- | --- | --- | --- | --- | --- | --- | --- | --- | --- |
| **Variable** | **M** | **SD** | **M** | **SD** | ***t*** | **df** | ***p*** | ***d*** |  |
| ***MAIA-2 (score range: 0–5)*** | | | | | | | | | |
| Noticing *[Sensing]* | 2.67 | 1.22 | 3.00 | 1.17 | *-1.582* | 116.5 | .116 | *-0.274* |  |
| Not-Distracting *[Attention]* | 2.39 | 1.21 | 2.46 | 0.95 | *-0.408* | 129.8 | .684 | *-0.068* |  |
| Not-Worrying *[Interpretation]* | 2.05 | 1.12 | 2.34 | 1.07 | *-1.502* | 116.8 | .136 | *-0.260* |  |
| Attention Regulation *[Attention]* | 1.94 | 1.16 | 2.48 | 1.12 | *-2.734* | 116.0 | **.007** | *-0.475* | **†** |
| Emotional Awareness *[Interpretation]* | 2.68 | 1.36 | 2.94 | 1.40 | *-1.104* | 110.6 | .272 | *-0.194* |  |
| Self-Regulation *[Attention]* | 1.70 | 1.02 | 2.10 | 1.34 | *-1.856* | 92.1 | .067 | *-0.344* |  |
| Body Listening *[Interpretation]* | 1.74 | 1.22 | 2.46 | 1.39 | *-3.123* | 102.9 | **.002** | *-0.561* | ***** |
| Trusting *[Interpretation]* | 1.97 | 1.41 | 2.66 | 1.26 | *-2.986* | 121.8 | **.003** | *-0.510* | * |
| ***DES-II (score range: 0–100)*** | | | | | | | | | |
| Total score | 29.04 | 18.12 | 19.97 | 13.70 | *3.327* | 131.9 | **.001** | *0.549* | ***** |
| Amnesia | 16.25 | 16.91 | 13.20 | 12.87 | *1.195* | 131.6 | .234 | *0.197* |  |
| Absorption/Imagination | 45.06 | 21.38 | 30.37 | 17.69 | *4.369* | 127.3 | **< .001** | *0.734* | ***** |
| Depersonalization/Derealization | 22.12 | 22.68 | 13.28 | 15.41 | *2.715* | 134.7 | **.007** | *0.439* | **†** |
| ***MDS-16 (score range: 0–100)*** | | | | | | | | | |
| Total score | 73.14 | 15.20 | 31.59 | 22.79 | *11.802* | 83.6 | **< .001** | *2.240* | ***** |
| Somato-sensory retreat | 71.94 | 19.13 | 42.59 | 22.31 | *7.950* | 100.9 | **< .001** | *1.436* | ***** |
| Interference with life | 74.34 | 15.88 | 20.58 | 26.74 | *13.325* | 77.5 | **< .001** | *2.581* | ***** |
|  | | | | | | | | | |

MD = maladaptive daydreaming group (n = 83); Control = control group (n = 54). All comparisons used Welch’s t-test. Degrees of freedom (df) are Welch–Satterthwaite values and are therefore non-integer. p values are two-tailed exact values. Cohen’s d was computed using the pooled standard deviation formula; positive values indicate higher scores in the MD group relative to controls. A Bonferroni correction was applied to control for familywise error rate across 13 comparisons, yielding an adjusted significance threshold of α = .003 (.05/15). MAIA-2 subscale scores range from 0 to 5; higher scores indicate greater endorsement of the respective interoceptive tendency. DES-II and MDS-16 scores range from 0 to 100; higher scores indicate greater dissociative symptomatology and greater MD severity, respectively. * Significant at Bonferroni-corrected α = .003. Attention Regulation (p = .007) and Depersonalization/Derealization (p = .007) did not survive Bonferroni correcti

# **Supplementary Table 4**. Edge-weight matrix extracted from the EBICglasso network

| Variable | 1 | 2 | 3 | 4 | 5 | 6 | 7 | 8 | 9 | 10 | 11 | 12 | 13 |
| --- | --- | --- | --- | --- | --- | --- | --- | --- | --- | --- | --- | --- | --- |
| 1. Som-Sen Ret |  |  |  |  |  |  |  |  |  |  |  |  |  |
| 2. Int with Life | 0.503 |  |  |  |  |  |  |  |  |  |  |  |  |
| 3. Amn | 0.000 | 0.000 |  |  |  |  |  |  |  |  |  |  |  |
| 4. Abs | 0.127 | 0.147 | 0.324 |  |  |  |  |  |  |  |  |  |  |
| 5. Dep/Der | 0.000 | 0.000 | 0.227 | 0.422 |  |  |  |  |  |  |  |  |  |
| 6. Notice | 0.000 | 0.000 | 0.000 | 0.000 | 0.000 |  |  |  |  |  |  |  |  |
| 7. Not-Dis | 0.000 | 0.000 | -0.091 | -0.042 | -0.091 | 0.000 |  |  |  |  |  |  |  |
| 8. Not-Wor | -0.011 | 0.000 | 0.000 | 0.000 | 0.000 | 0.000 | 0.000 |  |  |  |  |  |  |
| 9. AtReg | 0.000 | 0.000 | 0.000 | 0.000 | 0.000 | 0.064 | 0.000 | 0.000 |  |  |  |  |  |
| 10. EmAware | 0.000 | 0.000 | 0.000 | 0.024 | 0.000 | 0.234 | 0.000 | 0.000 | 0.126 |  |  |  |  |
| 11. Self-Reg | -0.030 | 0.000 | 0.000 | 0.000 | 0.000 | 0.020 | 0.000 | 0.071 | 0.232 | 0.115 |  |  |  |
| 12. Body List | 0.000 | -0.004 | 0.000 | 0.000 | 0.000 | 0.233 | 0.000 | 0.000 | 0.078 | 0.197 | 0.263 |  |  |
| 13. Trust | 0.000 | -0.107 | 0.000 | 0.000 | 0.000 | 0.024 | 0.000 | 0.026 | 0.192 | 0.000 | 0.104 | 0.180 |  |
